# Lessons Learned during Dengue Outbreaks in the United States, 2001–2011

**DOI:** 10.3201/eid1804.110968

**Published:** 2012-04

**Authors:** Amesh A. Adalja, Tara Kirk Sell, Nidhi Bouri, Crystal Franco

**Affiliations:** University of Pittsburgh Medical Center, Pittsburgh, Pennsylvania, USA

**Keywords:** dengue, vector control, mosquito, public health, outbreak response, viruses, vector-borne diseases, United States

## Abstract

Since 2001, three autochthonous dengue fever outbreaks have occurred in the United States: in Hawaii (2001); Brownsville, Texas (2005); and southern Florida (2009–2011). We sought to characterize and describe the response to these outbreaks from the perspectives of public health and vector control officials. By conducting a medical literature review through PubMed and news media searches through Google, we identified persons involved in managing each outbreak; 26 persons then participated in qualitative, semistructured interviews. After analyzing the 3 outbreaks, we found the following prominent themes in the response efforts: timely detection of illness; communication of up-to-date, correct information; and development of a rapid response that engages the community. We therefore recommend that public health authorities involve the clinical and laboratory community promptly, provide accurate information, and engage the local community in vector control and case identification and reporting.

## Abstract 2

Public health authorities should involve the clinical and laboratory community and engage the local community in vector control and case reporting.

Dengue is a mosquito-borne viral disease, endemic to tropical regions. In the United States, most dengue infections have been limited to travelers returning from dengue-endemic regions; the last outbreak in the continental United States occurred in 1945 (*1*). However, epidemic dengue remains a threat to US areas that have competent mosquito vector populations and host large numbers of travelers from dengue-endemic regions, as evidenced by the return of dengue to Florida (*1*).

Practical experience with dengue in the United States is decades old, and mitigation measures used decades ago may not be fully applicable today. As the threat of dengue grows, the risks for an outbreak and the responses needed must be understood.

In this article, we describe the responses to 3 recent US dengue outbreaks (in Hawaii, 2001; Brownsville, Texas, 2005; and southern Florida, 2009–2011) from the perspectives of public health and vector control officials at local, state, and federal levels. We conducted a retrospective analysis to assess mitigation strategies used during each outbreak and identify policy implications for public health departments, vector control agencies, and clinicians in areas vulnerable to dengue and other mosquito-borne diseases. The goal of this study was to help improve community responses to future dengue outbreaks. The analysis concludes with recommendations for practitioners and policy makers.

## Methods

To understand the outbreaks and identify contacts involved in managing each outbreak, the research team reviewed the medical literature through PubMed and also searched Google to identify names of outbreak management officials. Researchers then asked these persons to participate in qualitative, semistructured interviews. Additional participants were also identified and added throughout the interview process. In total, 26 persons were interviewed (9 from the Hawaii outbreak, 10 from the Florida outbreak, and 7 from the Texas outbreak). The interviewees from each outbreak included heads of local health departments, personnel from the US Centers for Disease Control and Prevention (CDC), vector control officers, and state and local health department staff.

During the interviews, the research team posed open-ended questions aimed at eliciting an understanding of how each outbreak was discovered and the outbreak management techniques, case-finding methods, and public outreach/engagement strategies that were used (Technical Appendix). In addition, questions were posed regarding the usefulness of specific interventions, including the interactions between city, county, state, and federal public health authorities, and outreach to physicians. As a final question, each interview participant was asked to describe what he or she would have done differently or what activities he or she would recommend for a future outbreak.

## Outbreak Findings

### Hawaii, 2001

#### Initial Case

The Hawaii outbreak was discovered in September 2001 by a non-island physician temporarily employed in the rural region of Hana on Maui. Before 2001, autochthonous dengue infections had last been definitively reported in Hawaii in 1944. (Two suspected cases were reported to have occurred in German travelers to Hawaii in 1995 [*2*]. In March 2011, however, 4 cases of dengue were confirmed on Oahu. Although travel-related dengue is not an uncommon diagnosis in Hawaii, these cases were acquired locally [autochthonous] [*3*].) Therefore, dengue was not considered in the differential diagnosis for persons without a travel history who sought treatment. The physician made a clinical diagnosis on the basis of the initial patient’s symptoms and alerted the Hawaii Department of Health, prompting an investigation that uncovered additional suspected autochthonous cases. However, laboratory confirmation was delayed because of the events of September 11, 2001, after which air travel was suspended and specimens could not be shipped to CDC. The outbreak was not officially confirmed until September 21, 2001 (*4*).

#### Outbreak

In all, 122 laboratory-confirmed cases were identified through 2002 (92 on Maui, 26 on Oahu, and 4 on Kauai). All isolates were typed as dengue virus type 1 (DENV-1) and had a specific envelope glycoprotein sequence, indicating that the strain was likely imported by travelers from French Polynesia, where a large DENV-1 epidemic caused by the same genotype was occurring (*4*). This outbreak was unique because it involved the less competent dengue mosquito vector *Aedes albopictus* (some interviewees speculated that this may have caused the outbreak to end relatively quickly). Cases began in a rural region of Maui, and subsequent cases were centered in areas with thick vegetation and heavy precipitation. Infections on the other islands represented local transmission. In 1 study, case-patients were found to be ≈7 times more likely to live in homes with birds, chickens, or both, than in homes without these animals (*5*). The response was managed at the state level, with state district health officials in the chain of command. CDC provided technical assistance. Interviewees noted that during the outbreak, tension existed between responding parties over jurisdictional issues that largely remained unresolved.

In addition to the epidemiologic and vector control response to the outbreak, officials also had to address issues that were politically and publicly sensitive. Because the Hawaii economy depends on tourism, the response had to balance the need for protective action on the part of local residents and tourists with the need to avoid discouraging tourism. Additionally, although some members of the public were concerned about the negative effects of pesticide use, others demanded that spraying be conducted around schools (which had questionable utility in combating the outbreak). Finally, community engagement practices had to be tailored to the needs of specific localities. For example, on 1 island, attendance at town hall meetings was high, but on another island, attendance at similar meetings was low. However, public health officials believed that residents of this second island were more receptive to receiving information from fliers distributed in general stores.

#### Mitigation and Response

After dengue cases in Maui were discovered, state health department officials began an aggressive campaign of public engagement involving town hall meetings, door-to-door campaigns to identify case-patients and educate the public about mosquito abatement, and media messaging (television, radio, and Internet). A public relations agency was hired to help manage questions from the public. The state public health department announced daily case counts at press conferences, and highway checkpoints were established for distribution of mosquito repellent. In addition, health officials engaged car rental agencies and hotels to distribute educational brochures for travelers and tourists.

Upon request, CDC deployed Epidemiologic Intelligence Service officers as well as vector experts to Hawaii. *Ae. albopictus* mosquitoes were soon found to be propagating the outbreak. Vector control activities included spraying to kill adult mosquitoes (adulticiding) within a 200-m radius around homes of case-patients, breeding-site control activities such as trash collection and elimination of standing water, and door-to-door campaigns to educate the public about eliminating mosquitoes around homes.

Persons who were interviewed emphasized that during vector control activities, they focused on addressing mosquito breeding sites and not on potential building code violations or the farming of prohibited plants. Outreach to clinicians included grand rounds presentations, visits with clinicians, and encouragement and support for clinicians to conduct testing of suspected case-patients.

An unpublished communications study was conducted by the Hawaii Department of Health during the outbreak to assess the general public’s response to key public health messages. Some key conclusions from this study included the finding that 16 (40%) of 90 residents surveyed stated that they took action to prevent dengue. Of those who took action, 74% eliminated stagnant water outside their homes and 63% took action to prevent mosquitoes from entering their homes (Hawaii Department of Health, unpub. data). Conclusions drawn from Hawaii’s response to the outbreak are listed in the Table.

**Table:** Lessons learned during US dengue outbreaks, 2001–2011*

Location, year	Lessons learned
Hawaii, 2001	Populations are not completely homogeneous, and messages should be tailored to specific locales.
	Tourism concerns must be balanced with public health response.
	Community engagement activities are palatable to the public when nonpunitive, actionable initiatives are undertaken by public health agencies.
	A communication study validates the community engagement approach, with substantial numbers of residents aware of the outbreak and those taking actions performing the correct action.
	A lack of in-state testing capacity delays confirmation of the outbreak.
	Although the *Aedes albopictus* mosquito is a competent vector, its involvement may limit this outbreak in a rural Hawaii setting, especially with prompt outbreak control efforts.
Brownsville, Texas, 2005	Nearby foci of endemicity make dengue a continual threat, including the possibility of dengue hemorrhagic fever.
	Involving CDC/BIDS facilitates fast identification of the index case.
	Pre-outbreak awareness of and preparation for the potential threat of dengue enhances the ability to respond to an actual outbreak.
Florida, 2009–2011	An aggressive multimodal campaign engages the public.
	Door-to-door vector control activities are essential; the ability to inspect property without homeowner permission improves coverage.
	Clear communication with tourism officials diminishes the possibility of opposing viewpoints.

*CDC, Centers for Disease Control and Prevention; BIDS, Border Infectious Disease Surveillance.

### Brownsville, Texas, 2005

#### Initial Case

In July 2005, a diagnosis of dengue hemorrhagic fever (DHF) was made for a woman who had become ill with symptoms consistent with dengue in June. She had traveled from Brownsville to Mexico for treatment and received a clinical diagnosis of dengue in Mexico. She returned to the United States and was hospitalized as symptoms progressed. At that time, although she was given a diagnosis of murine typhus in Texas, doctors conducted serologic testing for dengue virus. The collection of blood samples was facilitated by CDC’s Border Infectious Disease Surveillance project in conjunction with other CDC programs. The woman had no history of travel to Mexico in the 2 months before her illness, and her current dengue infection (presumably not her first, given the occurrence of DHF) had occurred in Texas (*6**,**7*).

#### Outbreak

Limited outbreaks of locally acquired dengue have occurred sporadically since 1980 in areas of Texas that border Mexico (*7**,**8*). During the 2005 Brownsville outbreak, 25 cases of dengue were found, 3 autochthonous cases and 22 in persons who had traveled to Mexico. This outbreak was part of an epidemic that included 1,251 cases of dengue in the bordering Mexican state of Tamaulipas during August 2005 (*7*). The outbreak was managed by using city, county, and state resources; CDC conducted Border Infectious Disease Surveillance project work and serosurveys. Laboratory testing for cases was performed by the state department of health. Serosurveys indicated evidence of recent dengue virus infection in 4% of the population of Brownsville (*6*). In a risk factor analysis, Brownsville residents with properties smaller than the median lot size were 15 times more likely to be seropositive for dengue, whereas non–US-born residents were 3 times more likely to be seropositive (*6*).

#### Mitigation and Response

Because of the risk of acquiring dengue in the regions of Texas bordering dengue-endemic Mexico, officials at the Texas State Department of Health had conducted a series of workshops in 2004 to develop mitigation and response tools, including plans for community clean-up days as well as a school play to educate schoolchildren about dengue. After the initial case was identified in 2005, health officials expanded case-finding activities through direct contact with clinicians, medical record reviews, and serosurveys.

Once the outbreak was confirmed, health officials undertook additional education efforts, including town hall meetings, visits to physicians’ offices, and media messaging (including the media in Mexico). The main thrust of their efforts was to increase discovery, diagnosis, and reporting of cases. This effort included facilitating collection of blood samples and testing them at the state health department. Increased vector control activities, including sampling of mosquitoes for dengue virus, were also conducted. However, some interviewees questioned the use of the latter because it was thought to divert resources and have poor predictive power. Health officials stressed that in areas of Brownsville with clusters of “fevers of unknown origin” (possibly representing unrecognized dengue cases), vector control was crucial.

Currently, Brownsville ranks dengue as a top priority and maintains ongoing efforts to combat it, including reducing mosquito breeding sites and increasing public awareness of dengue symptoms through a federal Environmental Protection Agency grant (e.g., encouraging use of reusable shopping bags with dengue information, direct mailings, and television interviews with health authorities).

The Brownsville outbreak also highlights the need for ongoing surveillance for vector-borne diseases, especially as decreases in funding for these activities are anticipated. The conclusions we drew from Brownsville’s response are listed in the Table.

### Florida, 2009–2011

#### Initial Case

In September 2009, a physician in New York notified Florida’s Monroe County Health Department of a diagnosis of dengue fever in a traveler returning from Key West (the patient had not traveled to other locations), heralding the first autochthonous dengue case in Florida since 1934. After identification of the first case, enhanced case-finding activities uncovered more autochthonous cases in Key West (*1*).

#### Outbreak

Ultimately, in Key West, Monroe County, 90 cases were identified as part of the outbreak (27 cases in 2009 and 63 in 2010) (*1**,**9*). A 2009 serosurvey indicated that 5.4% of Key West residents had evidence of recent dengue virus infection (*1*). In 2010–2011, autochthonous dengue fever was also discovered in 5 other Florida counties: Broward (1 case), Hillsborough (1 case), Martin (1 case), Palm Beach (2 cases), and Miami-Dade (3 cases). In at least 2 of these instances, the dengue serotype recovered was distinct from the Monroe County serotype, indicating >1 introduction of dengue into Florida (*9**–**11*). Interviewees from Key West stated that their cases were centered in the Old Town area of Key West, a tourist area and where the so-called Key West lifestyle is common. Interviewees stated that this lifestyle, which involves spending a high proportion of time outdoors and keeping house windows open, was thought to be responsible for the transmission of the virus.

#### Mitigation and Response

At the time of discovery of the outbreak, the priority of Monroe County Health Department was to prevent deaths from dengue. The health department began a campaign in collaboration with the Florida Keys Mosquito Control District to control the outbreak. Response strategies used in Key West included town hall meetings, door-to-door visits/inspections, a public information telephone line, tourism council press releases, editorials, dispatching a biologist to schools, and the use of a television program (called Mosquito TV). Outreach to clinicians was performed through visits from health department personnel. Interviewees also stated that a frank discussion with tourism officials was held, which created an environment in which public health, vector control, and tourism officials could work together. Many of the materials and response activities developed by Monroe County were used in other counties in Florida that experienced dengue cases.

Vector control activities focused on door-to-door visits at residences to assess the prevalence of mosquito breeding sites so authorities could intervene if mosquitoes were found. When breeding sites were discovered, residents were asked to participate in inspections of their property and were instructed on how to eliminate breeding sites. In some cases, when properties proximate to dengue case-patients were not easily accessible (because of a resident’s absence), vector control officers had to scale fences. Although Florida law sanctions these actions and authorizes fines for those who hinder mosquito control, officials did not issue fines. Vector control officials reported that often the same properties had repeat violations, highlighting the difficulties in changing behavior. In Florida, funding for vector control activities varies at the county level; for example, Monroe County is funded by a dedicated property tax (which covers vector as well as nuisance mosquitoes), while Broward and Miami-Dade vector control activities are funded through general funds. The variance in funding sources for vector control efforts affects the annual amount of funding allocated because budgets may allocate funds for vector control differently each year.

**Figure 1:**
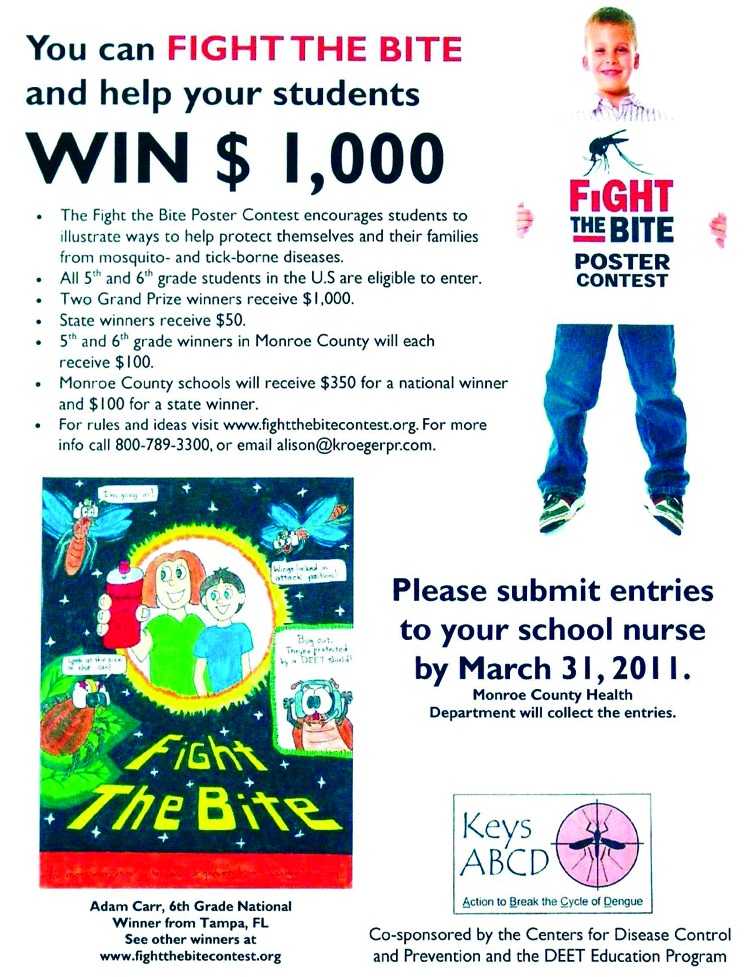
Example of an activity to engage the public in controlling dengue outbreaks, Florida, USA, 2009–2011.

**Figure 2:**
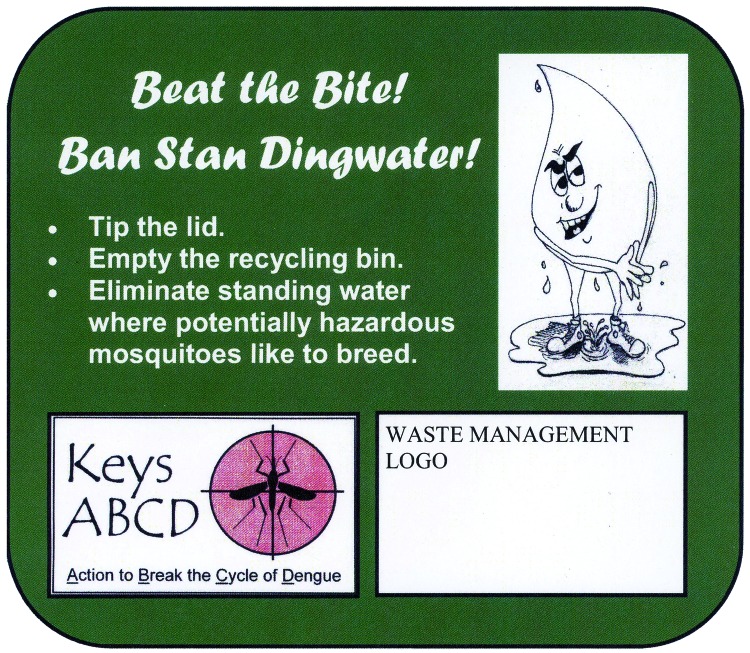
Cartoon character used in public relations campaign to control dengue outbreaks, Florida, USA, 2009–2011.

In January 2011, the Monroe County Health Department launched an initiative called ABCD (Action to Break the Cycle of Dengue), which is designed to “draw more members of the public into the fight against” dengue. The program has performed such activities as encouraging cemeteries to dump standing water, a “Fight the Bite” poster contest, continued posting of door hangers with dengue information, neighborhood cleanups of mosquito breeding sites, and creating cartoon characters to communicate information about dengue to children (and adults) (Figure 1, Figure 2). Conclusions drawn from Florida’s response to the outbreak are listed in the Table.

## 3 Key Recommendations

### Involve Clinical and Laboratory Community Promptly

First, we recommend that state and local public health agencies in areas at risk for autochthonous dengue engage the clinical community and develop capacity to better ensure prompt clinical diagnosis and laboratory detection of this disease. Early recognition and identification of dengue, a nationally reportable disease since 2010, are critical for successful response. In 2 of the outbreaks (Hawaii and Florida), clinicians from outside the outbreak area made the diagnosis. After suspected dengue cases were reported, local health department and mosquito control officials were able to act on this information to initiate their response.

These scenarios highlight the value of physician awareness of the signs, symptoms, and diagnostic testing related to dengue, especially in areas most at risk for autochthonous dengue (those areas with 1 or both of the competent mosquito vectors and a population of travelers from disease-endemic regions). Public health departments should dedicate time and effort to engage the clinical community in issues related to dengue. Clinicians in areas at risk for dengue—as well as those in areas that receive travelers from dengue-endemic regions—should know the signs and symptoms of the disease and the requirements for laboratory testing and confirmation and should report suspected and confirmed cases to public health departments.

In areas at risk for dengue, laboratories capable of doing on-site testing should be identified beforehand, and plans for sample collection should be predetermined. In localities where testing is not available, alternative plans for rapid and efficient testing should be developed. This information should be disseminated. In Hawaii, testing was not available in any laboratory within the state, and confirmation of the outbreak was delayed because of the need to ship samples to CDC. In the other outbreaks, testing could not be done locally but was done at the state level. Interviewees stated that the ability to confirm dengue fever—which may be difficult to distinguish clinically from influenza—is hampered when access to testing is not available locally.

In these 3 outbreaks, public health laboratories conducted most testing. However, private laboratories also demonstrated the ability to perform dengue serologic testing (and in Florida were first to confirm dengue [*1*]), illustrating that laboratory capacity need not be solely the responsibility of public health authorities. In fact, commercial laboratory chains offer serologic testing, and an IgM serologic test (cleared by the US Food and Drug Administration) is available that will enhance the confidence of public health departments in the assay (*12*) because the test result will clearly reflect current infection. However, infection with other flaviviruses may produce false-positive serologic results. In addition, IgM results can be negative in a proportion of samples from persons with secondary dengue infections (*13*). PCR and serotyping work would still require the use of CDC or university laboratories.

### Provide Accurate Information

Second, we recommend that public health agencies involved in responding to an outbreak of dengue commit themselves to providing accurate and up-to-date information to the public, other public health and vector control jurisdictions, policy makers, and the clinical community. During the 3 dengue outbreaks, communication flowed in many directions. Ensuring the flow of information among health jurisdictions, the public, and mosquito control personnel is essential for managing an outbreak. Because dengue is a mosquito-borne disease, it necessarily will encompass a wide variety of entities in outbreak management and inclusion of a more extensive group of stakeholders than a disease not involving a vector.

Once dengue is detected, no delay should occur in telling the public about the outbreak or in disseminating strategies to minimize risk. Open communication will enhance public trust and make persons and communities more likely to participate in response and mitigation activities. In Hawaii, daily press conferences were critical to updating the public on the status of the outbreak. In other outbreaks, using door hangers and other media to relay information about dengue was instrumental. Honest communication, tailored to public needs and to the ways that a population best receives information, is the most effective way to gain the public’s trust and cooperation in outbreak response (*14*). Public messaging should also provide specific actions that members of the public can take to protect themselves, their families, and their communities.

Interagency communication to political leadership about the risks and benefits of action is also an integral part of dengue outbreak response. Although, in general, relations between agencies and political leadership were constructive in each of the 3 outbreaks analyzed here, delayed initiation of coordination between government entities occasionally expended valuable time or led to contradictory public messages. Local public health and mosquito control agencies in areas with competent vector mosquito species should establish lines of communication with one another, with local and state governments, and with CDC before an outbreak. Concerns about the effects on tourism should be considered but should not interfere with effective public health management.

### Engage Affected Community in Vector Control, Case Identification, and Case Reporting

Third, we recommend that the public health response to an outbreak of dengue in the United States focus on engaging the affected community in vector control activities, case identification, and case reporting. The chief means of combating dengue is reducing mosquito populations. Given that dengue mosquito vectors are peridomestic and have breeding sites close to human dwellings, often in backyards, public engagement in mosquito abatement is essential for controlling a dengue outbreak (*15*). Each of the 3 US outbreaks mobilized the public to combat mosquitoes. The door-to-door efforts of mosquito control and public health personnel to educate residents and facilitate their engagement in the fight against dengue were deemed by interviewees as some of the most effective mitigation activities undertaken to control the outbreaks. All localities noted that one-on-one contact with the public played a key role in their outbreak response efforts. These activities will still require population-level evaluation to fully validate their effectiveness, however. Other mosquito abatement activities, such as aerial spraying, were often considered superfluous by interviewees.

Public health and vector control officials should engage directly with residents to identify case-patients and remove mosquito breeding sites from their properties. Simple tasks such as dumping standing water are essential and easy to perform. In addition, the focus of response should be on community engagement measures to control dengue, rather than on punitive measures (i.e., pest control citations or citations for other code violations). A positive approach to public engagement for dengue response will help avoid conflict with residents, will make residents more apt to participate in mosquito control and case reporting, and will build trust with local public health officials. An integrated response directed by vector control and public health officials that melds with community efforts may be the optimal approach (*16*).

## Supplementary Material

Technical Appendix Interviews were approximately 30–45 minutes and audio-recorded, when permitted by the interviewee.
