# Supplementary material for: Lessons Learned during Dengue Outbreaks in the United States, 2001–2011
Source: Emerg Infect Dis. 2012 Apr;18(4):608–14. doi: 10.3201/eid1804.110968 (PMC3309700; doi:10.3201/eid1804.110968)
Supplement: Technical Appendix — Interviews were approximately 30–45 minutes and audio-recorded, when permitted by the interviewee. [file 11-0968-Techapp_4p.pdf]

Article DOI: http://dx.doi.org/10.3201/eid1804.110968

Lessons Learned during Dengue Outbreaks in the United States, 2001–2011

Technical Appendix

Interview Questions

Interviews were approximately 30-45 minutes and audio-recorded, when permitted by the interviewee. All members of the interview team reviewed the questions and used the same questions for each interviewee. Answers were reviewed by all members of the research team. No discrepancies on matters of fact were noted amongst the interviewees, though minor interpretive differences existed.

Background

1. Tell me about yourself and your agency/clinic/organization?

Your position?

Your institution/organization's mission?

Management of Outbreaks

1. How did officials first become aware that dengue was in your community? How were cases first confirmed?

2. Was there more broad communication between the medical community and public health before the outbreak occurred? Was awareness of the outbreak disseminated between the two groups? (If yes, how?)

3. Did formal systems (i.e., public health, labs, hospitals, etc.) function the way you expected them to?

[If no]:

a. What contributed to that disconnect?

4. What was the community's reaction to a dengue outbreak?

5. Generally, at the time of the outbreak, how many clinicians were aware or knowledgeable of dengue, its symptoms, and diagnosis and treatment options?

6. What facilitated timely detection and confirmation of dengue illness? Can you describe the process (for clinicians, state and local laboratories, health departments, etc.) to detect and confirm dengue illness?

a. Are you aware of any situations in which patients experiencing symptoms of dengue did not present to a medical provider for evaluation? Why?

b. Are you aware of any issues hindering timely response and reporting of cases?

c. Are you aware of any issues of underreporting of dengue cases? If so, what factors contributed to underreporting?

d. Are you aware of any instances when cases were not reported? Why?

7. What types of interventions/efforts took place in [reference location] to mitigate the spread of dengue once cases were confirmed?

a. Can you describe the interventions/efforts that took place and who was involved? Specifically, what role did public health, clinicians, and vector control play in these interventions?

b. Were these interventions/efforts useful and successful? Why or why not?

[If none mentioned]:

a. Why do you think no interventions were taken?

8. Would you deem the public health and medical response to this outbreak to have been adequate? What could have strengthened the response of state/ local health departments, hospitals, and clinicians (i.e., staffing, resources, etc.)?

9. If a dengue outbreak were to happen again, would the county/state be able to respond in a timely manner? What could enhance response efforts (information, resources, etc.)?

10. What could officials at the state and local level do in the future to increase timely management and response to outbreaks of dengue and other vector-borne diseases?

Institutional Relationships

1. What presence do vector control programs have in your district/region? To the best of your ability, can you describe the interactions between public health and medical communities and vector control/mosquito abatement programs?

Community Engagement and Mitigation Strategies

1. Prior to the outbreak, are you aware of any efforts between public health, vector control, or the clinical communities to educate the public and/or the media about dengue?

[If yes]:

a. Can you describe who communicated with the public and how?

[If no]:

b. Why do you think dengue was not addressed with the public?

2. During the outbreak, were there any attempts to either educate the public about clinical aspects of dengue and/or the role of vector control programs in disease prevention and mitigation?

[If yes]:

a. Can you describe who communicated with the public and how?

b. How was the public educated about the signs and symptoms of dengue or when they should present to a medical provider for testing?

c. How was the public educated about the importance of vector control? Was there knowledge of the role of mosquito abatement in mitigating spread of dengue?

[If no]:

a. Why do you think dengue was not addressed with the public?

3. What was the role of the media in educating the public about the outbreaks? In educating communities about how to mitigate spread?

a. How did public health and clinicians work with the media?

b. What could the media do to increase awareness about dengue and the role of vector control?

4. How well do you think communication efforts went during the outbreak? Were messages well received by the public? Were there changes in behavior?

5. If an outbreak happened again, how could communication with the public be improved to involve them in mitigation efforts?

Policy Suggestions

1. If a dengue outbreak happened again, and was worse than what previously occurred:

a. What major challenges would [healthcare, public health, vector control] face?

b. What could policymakers do to address some of these challenges?*

**Note*: make this question specific according to who we are talking to— i.e. if a local health department official, ask about recommendations to the state health department.

2. What role do you think the CDC should play with state and local health departments, hospitals, and vector control to prevent, mitigate and respond to dengue outbreaks?

Conclusion

1. Is there anything else you would like to add?

2. Do you have any leads/suggestions of others we should talk to?

3. Would you like to be added to our list to receive our report findings?
